# Supplementary figures and images for: Relationship between diabetic macular edema and choroidal layer thickness
Source: PLoS One. 2020 Jan 7;15(1):e0226630. doi: 10.1371/journal.pone.0226630 (PMC6946145; doi:10.1371/journal.pone.0226630)

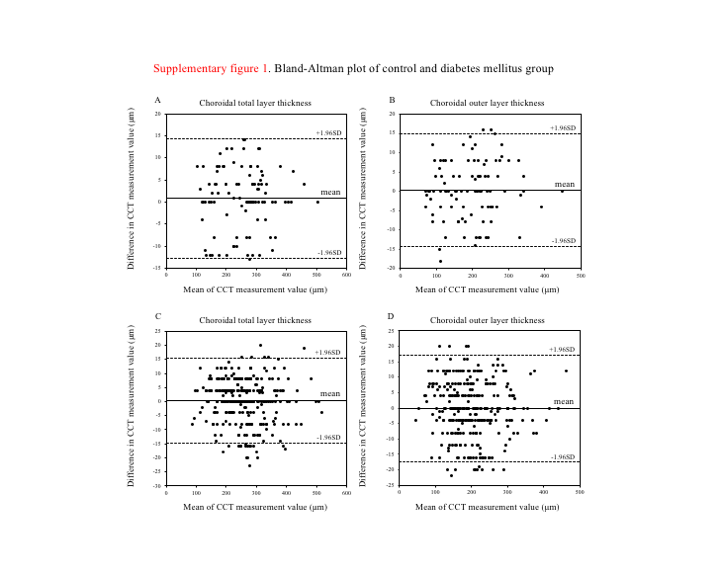

Supplement: S1 Fig — Total choroidal thickness (A) and outer choroidal thickness (B) in normal eyes. Total choroidal thickness (C) and outer choroidal thickness (D) in diabetic eyes. Solid line indicates the average mean difference, while dotted line shows 95% confidence limit of agreement. There is no specific trend to result in the difference between raters. (TIFF) [file pone.0226630.s001.tiff]
