# Supplementary material for: Relationship between diabetic macular edema and choroidal layer thickness
Source: PLoS One. 2020 Jan 7;15(1):e0226630. doi: 10.1371/journal.pone.0226630 (PMC6946145; doi:10.1371/journal.pone.0226630)
Supplement: S1 Table — (DOCX) [file pone.0226630.s003.docx]

**S1 table. Inter-examiner reliability of choroidal segmentation for control and diabetes mellitus group**

|  |  |  |  |  | Bland-Altman analysis | | | | |
| --- | --- | --- | --- | --- | --- | --- | --- | --- | --- |
|  |  | Relative reliability | |  | Fixed bias | |  | Proportional bias | |
|  |  | SCC | *P* value |  | CI 95% | *P* value |  | *r* | *P* value |
| Control group | Choroidal total layer thickness | 0.991 | ＜0.001 |  | -0.56~2.20 | 0.24 |  | 0.007 | 0.94 |
|  | Choroidal outer layer thickness | 0.987 | ＜0.001 |  | -1.22~1.74 | 0.73 |  | 0.099 | 0.33 |
| DM group | Choroidal total layer thickness | 0.984 | ＜0.001 |  | -0.44~1.27 | 0.34 |  | -0.109 | 0.053 |
|  | Choroidal outer layer thickness | 0.972 | ＜0.001 |  | -0.86~1.09 | 0.82 |  | -0.101 | 0.07 |

SCC, spearman's rank correlation coefficient; CI 95%, 95% confidence interval; DM, diabetes mellitus
